# Case Report: Severe SIADH and QTc prolongation induced by escitalopram-quetiapine interaction in a CYP2C19 intermediate metabolizer at therapeutic doses

**DOI:** 10.3389/fphar.2026.1776959

**Published:** 2026-01-30

**Authors:** Zongchen Jiang, Xiaoyu Qu, Zimin Yan, Jungang Fang, Jin Lan, Xin Zhao, Wensheng Qi

**Affiliations:** 1 Guang’anmen Hospital, China Academy of Chinese Medical Sciences, Beijing, China; 2 Department of Emergency, Guang’anmen Hospital, Beijing, China

**Keywords:** CYP2C19, drug safety, escitalopram, phenoconversion, QTc prolongation, SIADH

## Abstract

Escitalopram is widely regarded as a well-tolerated selective serotonin reuptake inhibitor (SSRI) with a favorable safety profile. However, severe adverse events can occur even at therapeutic doses in susceptible individuals. Here, we report a rare case of simultaneous life-threatening Syndrome of Inappropriate Antidiuretic Hormone secretion (SIADH) and cardiac toxicity induced by standard-dose escitalopram. A 51-year-old female (weight 50 kg) presented with severe fatigue and anorexia. Initial laboratory results revealed profound hyponatremia (116.1 mmol/L). Following sodium supplementation, serum sodium paradoxically decreased to 114.7 mmol/L (“desalination phenomenon”), while urinary sodium excretion was markedly elevated (220 mmol/24 h) alongside significant hypouricemia (76 μmol/L), confirming the diagnosis of SIADH. Concurrently, the patient manifested significant cardiac toxicity, including sinus bradycardia (41–55 bpm) and marked QTc prolongation (570 ms). Pharmacogenetic analysis identified the CYP2C19 *1/*2 genotype (Intermediate Metabolizer). Despite the therapeutic dosage (10 mg/day) and a non-toxic serum concentration (5 ng/mL measured 72 h post-discontinuation), the patient exhibited severe toxicity, likely driven by “phenoconversion” due to low muscle mass and physiological vulnerability, exacerbated by a pharmacodynamic synergism with low-dose quetiapine. Discontinuation of medications and strict fluid management resulted in complete resolution of both hyponatremia and arrhythmia. The causality was assessed as “probable” for both drugs using the Naranjo Algorithm, and the drug-drug interaction was rated as “probable” using the Drug Interaction Probability Scale (DIPS). This case highlights that genotype-phenotype mismatch, combined with pharmacodynamic synergism (escitalopram-quetiapine interaction), can precipitate severe neuro-cardiac toxicity even at therapeutic levels. It underscores that severe neuro-cardiac toxicity can occur even at therapeutic levels due to individual vulnerability. Therefore, routine monitoring of electrolytes and electrocardiograms (ECG) remains indispensable for patient safety, as pharmacogenetic screening and therapeutic drug monitoring may not predict such idiosyncratic reactions in resource-constrained settings.

## 1 Introduction

Selective serotonin reuptake inhibitors (SSRIs), particularly escitalopram, are widely prescribed as first-line treatments for depression and anxiety due to their generally favorable safety profile compared to tricyclic antidepressants (Peretti et al., 2000; Rief et al., 2009; Anderson, 2000; Cipriani et al., 2018; Cipriani et al., 2009). However, electrolyte disturbances, specifically hyponatremia secondary to the syndrome of inappropriate antidiuretic hormone secretion (SIADH), remain a significant complication. The incidence of SSRI-induced hyponatremia varies widely, ranging from 0.5% to 32%. Older adults are particularly vulnerable due to age-related decline in renal water excretion, low body weight, and polypharmacy (Liamis et al., 2008; Leth-Møller et al., 2016; Jacob and Spinler, 2006; Capinha et al., 2025). Drug-induced hyponatremia in this population is often overlooked but can lead to severe consequences, including cognitive impairment and falls.

While hyponatremia often presents as a metabolic emergency, emergency physicians must also remain vigilant regarding concurrent cardiovascular toxicity. Escitalopram is known to cause dose-dependent QTc interval prolongation, prompting specific regulatory warnings (US Food and Drug Administration, 2011; Kim et al., 2021; Hasnain et al., 2013; Ojero-Senard et al., 2017; Kim et al., 2016). Furthermore, the co-administration of atypical antipsychotics, such as quetiapine, may exacerbate this risk through pharmacodynamic synergism, potentially leading to life-threatening arrhythmias like torsade de pointes (TdP), especially in the presence of electrolyte abnormalities (Hasnain et al., 2014; Das et al., 2021; Matsuo and Yamaori, 2022).

The metabolic clearance of escitalopram is primarily mediated by the cytochrome P450 2C19 (CYP2C19) enzyme. Genetic polymorphisms in CYP2C19 significantly influence drug exposure and toxicity risk (Yu et al., 2003; Tsai et al., 2010; Chang et al., 2014; Jin et al., 2010; Noehr-Jensen et al., 2009; Jukić et al., 2018). While “poor metabolizers” (PM) are widely recognized as high-risk candidates for drug accumulation, the clinical implications for “intermediate metabolizers” (IM, e.g., *1/*2 genotype) are less frequently reported, particularly regarding severe adverse events at therapeutic dosages. In specific clinical contexts—such as low body weight or physiological decline—these patients may undergo “phenoconversion,” exhibiting a toxicity profile resembling that of poor metabolizers (Klomp et al., 2020; de Jong et al., 2023; Scherf-Clavel et al., 2024; Darakjian et al., 2021; Mostafa et al., 2021; Lenoir et al., 2021).

Here, we report a rare case of a 51-year-old female presenting to the Emergency Department with severe symptomatic hyponatremia (116 mmol/L) and significant QTc prolongation (570 ms). Despite receiving therapeutic doses of escitalopram and low-dose quetiapine, the patient developed life-threatening neuro-cardiac toxicity. This report details the diagnostic utility of the “desalination phenomenon” in confirming SIADH and explores the critical role of the CYP2C19 intermediate metabolizer genotype in the pathogenesis of toxicity at therapeutic levels.

## 2 Case description

A 51-year-old female (weight 50 kg, height 158 cm) presented to the Emergency Department on Day 0 with a 1-day history of worsening fatigue, nausea, and anorexia. Four months prior to admission, the patient underwent a comprehensive health check-up, including a head CT and 24-h Holter monitoring, which revealed no structural brain lesions and a normal baseline heart rate variability (SDNN 146 ms) without arrhythmia. Her medical history was significant for anxiety diagnosed 1 month prior, initially treated with citalopram 20 mg daily. This was switched 10 days prior to presentation to escitalopram 10 mg daily, and quetiapine 50 mg daily was simultaneously initiated, alongside zopiclone for insomnia. Upon arrival (Day 0), she was hemodynamically stable but hypertensive (blood pressure 168/96 mmHg) with a heart rate of 61 bpm. Initial laboratory evaluation revealed severe hyponatremia (serum sodium 116.1 mmol/L) with concomitant hypochloremia (83.1 mmol/L) and low-normal potassium (3.47 mmol/L). Following initial antiemetic treatment, an infusion of 0.9% saline supplemented with hypertonic saline was initiated to correct the hyponatremia (Table 1).

**TABLE 1:** Temporal evolution of laboratory parameters, vital signs, and therapeutic interventions.

Parameter (Normal Range)	Day 0 (Admission)	Day 1 (Worsening)	Day 2 (Diagnosis & sampling)	Day 6 (Discharge)
Intervention phase	Emergency visit	Saline infusion^a^	Drug discontinuation (72 h)	Recovery
Serum electrolytes				
Sodium (137–147 mmol/L)	116.1	114.7 ↓	132.8	135.2
Potassium (3.5–5.3 mmol/L)	3.47	4.53	4.03	3.76
Chloride (99–110 mmol/L)	83.1	86.8	104.1	100.5
SIADH markers				
Serum osmolality (275–305 mOsm/kg)	-	258 (hypotonic)	-	-
Urine osmolality (600–1,000 mOsm/kg)	-	432	-	-
Serum uric Acid (208–428 μmol/L)	-	76 (1.28 mg/dL)↓↓	-	131 (2.20 mg/dL)↑
Serum BUN (2.9–8.2 mmol/L)	-	1.8 ↓	-	-
Urine sodium	-	-	220 (24 h)^b^	-
Renal function				
Serum creatinine (44–133 μmol/L)	-	33	-	44
Cardiac & drug monitoring				
Heart rate (bpm)	61	41–55 (Bradycardia)	65	65
QTc Interval (ms)	-	570 (prolonged)	-	412 (normal)
Escitalopram concentration (ng/mL)	-	-	5.0	-
Medication status	Escitalopram 10mg, quetiapine 50 mg	STOPPED	Off-drug	Off-drug

^a^ Note the “Desalination phenomenon”: Serum sodium levels paradoxically decreased from 117.7 to 114.7 mmol/L following isotonic saline infusion, highly suggestive of SIADH.

^b^ 24-h urinary sodium excretion; Spot urine sodium was 58 mmol/L.

↓ indicates values significantly below the lower limit of normal.

Despite aggressive sodium supplement, repeat testing on the morning of Day 1 revealed a paradoxical decline in serum sodium to 114.7 mmol/L. This “desalination phenomenon”—where serum sodium drops following isotonic saline administration due to the rapid renal excretion of sodium while retaining free water—strongly suggested the Syndrome of Inappropriate Antidiuretic Hormone secretion (SIADH) rather than hypovolemic hyponatremia (Steele et al., 1997; Musch and Decaux, 1998; Sterns and Silver, 2008). Simultaneously, continuous cardiac monitoring alerted the clinical team to a new-onset sinus bradycardia (fluctuating between 41 and 55 bpm) and a markedly prolonged QTc interval of 570 ms (QTc-E 509 ms). Suspecting drug-induced neuro-cardiac toxicity (US Food and Drug Administration, 2011; Kim et al., 2021; Hasnain et al., 2013; Ojero-Senard et al., 2017; Kim et al., 2016; Hasnain et al., 2014; Das et al., 2021; Matsuo and Yamaori, 2022), a formal decision was made to permanently withhold escitalopram and quetiapine. Upon detailed history taking, it was revealed that the patient had already self-discontinued these medications on the day of symptom onset (Day −1) due to severe intolerance. Consequently, therapeutic management focused on strict fluid restriction and supportive care to facilitate drug washout, while sodium levels were carefully managed with hypertonic saline boluses.

A comprehensive diagnostic workup on Day 2 confirmed the diagnosis of SIADH. The patient was clinically euvolemic with a low serum osmolality (258 mOsm/kg) and inappropriately elevated urine osmolality (432 mOsm/kg). Crucially, the biochemical profile showed hallmark features of SIADH, including profound hypouricemia (serum uric acid 76 μmol/L), a low blood urea nitrogen (1.8 mmol/L), and massive urinary sodium excretion (220 mmol/24 h) (Fenske et al., 2008). Other etiologies were rigorously excluded: thyroid function (TSH 1.53 mIU/L, FT4 1.33 ng/dL) and adrenal function (ACTH 43.08 pg/mL, Cortisol 22.27 μg/dL) were within normal limits, and N-terminal pro-brain natriuretic peptide (NT-proBNP) was normal (<35 pg/mL), ruling out cardiac failure as a primary cause of hyponatremia.

**FIGURE 1:**
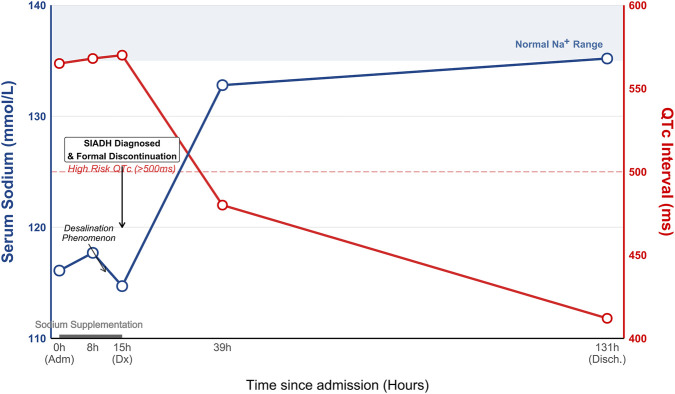
Timeline of the clinical course and relevant laboratory parameters. The left y-axis represents serum sodium levels (blue line), and the right y-axis represents the QTc interval (red line). Key events include saline infusion (Day 1), drug discontinuation (Day 2), and recovery (Day 6). Note the “desalination phenomenon” on Day 1 and the parallel improvement of hyponatremia and QTc prolongation after drug withdrawal.

Consistent with the progressive pharmacokinetic washout of the drugs and the implementation of strict fluid restriction, the patient’s clinical status improved markedly (Figure 1). By Day 3, serum sodium had normalized to 132.8 mmol/L, and the heart rate stabilized at 65 bpm. Pharmacogenetic testing revealed the patient to be a CYP2C19 intermediate metabolizer (*1/*2 genotype) (Bousman et al., 2023a; Hicks et al., 2017). Genetic testing identified the loss-of-function allele c.681G>A (*2), while c.636G>A (*3) and promoter variant c.-806C>T (*17) were wild-type. Therapeutic drug monitoring performed 72 h after the last dose showed a serum escitalopram concentration of 5 ng/mL (Schoretsanitis et al., 2018). By Day 6, the patient was fully recovered with a stable serum sodium of 135.2 mmol/L, normalized serum uric acid (131 μmol/L), and resolution of QT prolongation (QTc 412 ms). From the patient’s perspective, she reported a resolution of the “inner restlessness” and fatigue, expressing significant relief to learn that her symptoms were somatic consequences of the medication rather than a psychiatric deterioration. The causality of the adverse drug reaction was assessed as “probable” for both escitalopram (score 7) and quetiapine (score 6) using the Naranjo Probability Scale (Naranjo et al., 1981). Furthermore, the drug-drug interaction between escitalopram and quetiapine regarding QTc prolongation was evaluated as “probable” (score 7) using the Drug Interaction Probability Scale (DIPS) (Horn et al., 2007). At the 1-month follow-up after discharge, the patient had discontinued all psychotropic medications. She was managed with Traditional Chinese Medicine (TCM) and psychological counseling, reporting stable anxiety symptoms and no recurrence of hyponatremia or palpitations.

## 3 Discussion

The severe hyponatremia observed in this case resulted from the Syndrome of Inappropriate Antidiuretic Hormone secretion (SIADH), a well-documented but often underestimated adverse effect of serotonergic agents. The pathophysiology likely involves the overstimulation of central 5-HT2C and 5-HT7 receptors, which enhances the release of arginine vasopressin (ADH) and increases renal sensitivity to the hormone (Rottmann, 2007; Liu et al., 1996; Barclay and Lee, 2002). Clinically, this manifested in our patient not only as physical weakness but also as significant psychomotor agitation (described by the patient as “inability to sit still”), which is a hallmark of hyponatremic encephalopathy superimposed on serotonergic overstimulation (Tucci et al., 2020; Lane, 1998; Basu et al., 2014). While the initial clinical presentation mimicked dehydration, the diagnosis of SIADH was solidified by the characteristic “desalination phenomenon” observed on Day 1, where isotonic saline infusion paradoxically worsened the hyponatremia. This occurs because the kidney, under the influence of inappropriate ADH, excretes the infused sodium while retaining the free water (Steele et al., 1997; Musch and Decaux, 1998; Sterns and Silver, 2008). Furthermore, we utilized the diagnostic criteria established by Fenske et al. (Fenske et al., 2008), which suggest that hypouricemia and high fractional uric acid excretion are superior to serum parameters alone for distinguishing SIADH from volume-depleted states. Our patient’s profoundly low serum uric acid (76 μmol/L) and elevated urinary sodium (220 mmol/L) provided indisputable biochemical evidence of SIADH, ruling out hypovolemic etiologies.

A pivotal finding in this case is the discordance between the therapeutic dosage used (10 mg/day) and the severity of the toxicity, which can be elucidated by the patient’s pharmacogenetic profile. The patient carried the CYP2C19 *1/*2 genotype, classifying her as an Intermediate Metabolizer (IM). According to CPIC guidelines, IMs have reduced enzymatic activity, yet they are typically expected to tolerate standard starting doses (Bousman et al., 2023a; Hicks et al., 2017). However, clinical reality often diverges from genetic prediction due to “phenoconversion,” a phenomenon where an individual’s functional metabolic capacity is lower than their genotype suggests due to non-genetic factors (Klomp et al., 2020; de Jong et al., 2023; Scherf-Clavel et al., 2024; Darakjian et al., 2021; Mostafa et al., 2021; Lenoir et al., 2021; den et al., 2023). In this 51-year-old patient, low body weight (50 kg) and reduced muscle mass (indicated by a serum creatinine of 33 μmol/L) likely resulted in a smaller volume of distribution. However, given that the serum concentration was within the therapeutic range, the observed toxicity suggests a heightened pharmacodynamic sensitivity (individual vulnerability) rather than simple pharmacokinetic accumulation. A comparison with previously reported cases (Table 2) confirms that severe hyponatremia can precipitate even at low doses (e.g., 5 mg) (Kacperczyk et al., 2021; Prakash et al., 2021). In our case, the serum escitalopram concentration measured 72 h post-discontinuation was 5 ng/mL. Based on the elimination half-life of escitalopram (approx. 30 h), the back-calculated concentration at the time of admission would be approximately 20–30 ng/mL, which falls well within the consensus therapeutic reference range (15–80 ng/mL). This finding is pivotal: it confirms that the severe multi-organ toxicity was not caused by a supratherapeutic overdose, but rather represents “toxicity at therapeutic levels” driven by individual susceptibility. However, as notably shown in the table, most prior reports lack specific pharmacogenetic data or simultaneous cardiac safety monitoring. This distinguishes our case as a comprehensive documentation of how individual susceptibility—driven by the gene-environment interaction—outweighs dose magnitude as a predictor of toxicity.

**TABLE 2:** Review of reported cases of Citalopram/Escitalopram-induced SIADH: comparison of genotype availability and cardiac monitoring.

Author (Year)	Patient (Age/Sex)	Drug and Doseage	Lowest Na^+^ (mmol/L)	CYP2C19 Genotype	Cardiac monitoring (HR/QTc)	SIADH markers (U-Na/S-Uric Acid)
Barclay and Lee (2002) ^a^	87/M	Citalopram 20 mg	122	NR	HR 62 bpm (normal)	U-Na: 67 S-UA: Low
Tsai et al. (2012)	73/F	Escitalopram 10 mg	122	NR	NR	U-Na: 64 S-UA: NR
Kacperczyk et al. (2021)	85/F	Escitalopram 5 mg	123	NR	NR	Diagnosis by exclusion
	84/F	Escitalopram 5 mg	124	NR	NR	Diagnosis by exclusion
Prakash et al. (2021)	82/F	Escitalopram 5 mg	115	NR	Tachycardia (120 bpm)	U-Na: High^b^ S-UA: NR
Present case	51/F	Escitalopram 10 mg Quetiapine 50 mg	114.7	*1*/2 (IM)	Prolonged (570 ms) Bradycardia	U-Na: 220 S-UA: 76

Abbreviations: NR: not reported; IM: intermediate metabolizer; U-Na: Urine Sodium (mmol/L); S-UA: Serum Uric Acid (μmol/L); HR: heart rate.

^a^ Classic report reviewing earlier cases (e.g., Bouman 1998; Odeh 2001).

^b^ Qualitative description in text; exact value not provided.

The concurrent cardiac toxicity observed—severe sinus bradycardia and QTc prolongation—highlights a critical “double-hit” pharmacodynamic interaction. While escitalopram is known to cause dose-dependent QT prolongation, recent evidence also links it to profound sinus bradycardia and sinus arrest, particularly in patients with underlying cardiovascular risks (Li et al., 2024; Ge et al., 2025). In our patient, the QTc interval of 570 ms was likely the result of a synergistic blockade of the hERG potassium channel by both escitalopram and quetiapine. Although the quetiapine dose was low (50 mg), its combination with an SSRI was assessed as a “probable” interaction (DIPS score 7) (Horn et al., 2007). Quetiapine, metabolized by CYP3A4, may exert a pharmacodynamic synergistic effect with escitalopram on hERG potassium channel blockade. In a patient with reduced metabolic clearance can precipitate significant repolarization delays (Kim et al., 2016; Hasnain et al., 2014). Crucially, the pro-arrhythmic risk was amplified by the severe electrolyte derangements (hyponatremia) associated with SIADH (Tamene et al., 2010; Wu et al., 2021; Yalta et al., 2024; Yong et al., 2011). Although recent mechanistic studies (Wu et al., 2021) have highlighted the complex and variable role of sodium modulation in cardiac electrophysiology (e.g., in masking specific genetic phenotypes), profound hyponatremia is widely recognized in clinical practice as a destabilizing factor that can exacerbate drug-induced repolarization delays. This case reinforces that the CYP2C19 intermediate metabolizer status does not merely affect drug levels but can lower the threshold for multi-organ toxicity (neuro-cardiac) when multiple “minor” risk factors align.

In summary, this case challenges the assumption that therapeutic doses of SSRIs are intrinsically safe in patients without “poor metabolizer” genotypes. It demonstrates that severe SIADH and life-threatening arrhythmias can manifest simultaneously in CYP2C19 intermediate metabolizers, particularly when physiological reserves are diminished. Consequently, we advocate that for elderly or low-body-weight patients, who are already at high risk for drug-induced hyponatremia due to physiological decline and comorbidities (Capinha et al., 2025), particularly those requiring polypharmacy with potential cardiac risks, preemptive CYP2C19 genotyping and early therapeutic drug monitoring (TDM) should be considered to prevent such “unexpected” toxicity (Hiemke et al., 2018).

In conclusion, this case underscores a critical clinical reality: life-threatening neuro-cardiac toxicity can manifest even at standard therapeutic dosages of escitalopram when specific pharmacogenetic and physiological vulnerabilities align. The severe SIADH and significant QTc prolongation observed in this 51-year-old patient challenge the conventional perception of SSRIs as benign agents, illustrating how the CYP2C19 intermediate metabolizer status (*1/*2 genotype), compounded by low body mass and polypharmacy, can precipitate a functional “poor metabolizer” phenotype—a process known as phenoconversion. Furthermore, the concurrent use of low-dose quetiapine likely exerted a synergistic pharmacodynamic effect on cardiac repolarization, amplifying the arrhythmogenic risk created by the electrolyte derangements. Consequently, we advocate for a back-to-basics approach in the management of vulnerable populations. Since toxicity can manifest despite normal drug levels, clinical vigilance cannot rely solely on dosage guidelines or advanced testing; instead, early and rigorous monitoring of serum sodium and QTc intervals is essential. While TDM and pharmacogenetic screening are valuable tools for investigating complex cases (Pardiñas et al., 2021; Zanardi et al., 2021; Bousman et al., 2023b), routine biochemical and cardiac monitoring remain the most practical and effective strategies to prevent severe, unexpected adverse drug reactions in daily practice.

## Data Availability

The original contributions presented in the study are included in the article/supplementary material, further inquiries can be directed to the corresponding authors.
